# Severe hypercalcemia in a patient with chronic lymphocytic leukemia and non-small cell lung carcinoma: A case report

**DOI:** 10.1097/MD.0000000000024982

**Published:** 2021-04-09

**Authors:** Elena Chertok Shacham, Dafna Chap Marshak, Shay Brikman, Guy Dori, Avraham Ishay

**Affiliations:** a Department of Internal Medicine E, HaEmek Medical Center; b Endocrinology Unit, HaEmek Medical Center; c Hematology Unit, HaEmek Medical Center, Afula; d Faculty of medicine, Technion – Israel Institute of Technology, Haifa, Israel.

**Keywords:** case report, chronic lymphocytic leukemia, concurrent malignancies, hypercalcemia of malignancy, treatment of hypercalcemia, venetoclax

## Abstract

**Rationale:**

Hypercalcemia is a common finding in patients with advanced-stage cancers. Paraneoplastic hypercalcemia is commonly associated with dismal prognoses, with survival rates of about 3 months. In this paper, we report on a patient with advanced chronic lymphocytic leukemia and non-small cell lung carcinoma who developed severe hypercalcemia and discuss the diagnosis and treatment of this metabolic complication.

**Patient concerns:**

A 56-year old male with a 2-year history of Rai stage IV chronic lymphocytic leukemia presented with life-threatening hypercalcemia. Positron emission tomography/computed tomography revealed a suspicious lung lesion. A transbronchial biopsy was performed from the upper left lobe. Due to the small size of the specimen, immunohistochemical markers were performed and revealed positive staining for cytokeratin 7 and negative for TTF-1, napsin A and p 40, which were consistent with non-small cell lung carcinoma.

**Diagnosis:**

Humoral hypercalcemia of malignancy was diagnosed.

**Intervention:**

The patient was treated with saline infusion, calcitonin, intravenous pamidronate, followed with denosumab.

**Outcomes:**

The hypercalcemia was successfully treated and the patient's calcium levels returned to normal. Further evaluation revealed a non-small cell lung carcinoma as a second primary malignancy. The patient was treated with venetoclax for his refractory CLL and received chemotherapy and immunotherapy for lung adenocarcinoma. Several days after starting venetoclax, he developed Legionella pneumonia and short time after the second course of chemotherapy, a severe sepsis occurred and he passed away.

**Lessons:**

Coexistence of 2 unrelated malignancies, whichever could be a reason for hypercalcemia of malignancy is a rare event. Severe hypercalcemia, which is possible but rare feature of CLL should be a reason for further prompt evaluation.

## 1 Introduction

The estimated annual prevalence of hypercalcemia related to malignancy is 1.46% to 2.74%.^[1]^ Generally, patients with malignancy-related hypercalcemia have clinically overt cancer and markedly elevated serum calcium levels. It is 4 times more common in stage IV cancers and is associated with a poor prognosis. Solid tumors, like breast and lung cancers, and multiple myeloma are among the most common causes of cancer-induced hypercalcemia, which is generally linked to an increased circulation level of parathyroid hormone-related protein.^[2]^ Although a minority of hypercalcemic patients may present with hypercalcemia level >13.5 mg/dl hematologic malignancies including lymphoma, multiple myeloma, and small cell leukemia/lymphoma can lead to resistant hypercalcemia.^[2]^ Because of the enhancing effect of parathyroid hormone-related protein on osteoclastic bone resorption, antiresorptive therapy is a mainstay of the therapeutic approach to hypercalcemia associated with cancer.^[2,3]^ Recently, denosumab, a monoclonal antibody to receptor activator of nuclear factor kappa-B ligand (RANK) ligand, was found to be promising treatment for bisphosphonate resistant malignant hypercalcemia.^[2,3,4]^

Here we present a patient with advanced stage CLL, diagnosed with non-small cell lung carcinoma during evaluation for severe hypercalcemia. The coexistence of 2 different malignancies in the same patient, either of which could result in humoral hypercalcemia of malignancy is a challenging event. Only a very few cases of concurrent cancer-induced hypercalcemia events have been reported in the medical literature.

## 2 Case report

A 56-year old man presented to the Department of Internal Medicine with a deteriorated mental status. Laboratory tests performed in the emergency department demonstrated severe hypercalcemia at 17.95 mg/dl (reference range: 8.7–10.4), together with albumin levels of 3.2 g/dl, elevated levels of phosphor 5.9 mg/dl (reference range: 2.5–5), acute kidney injury with a creatinine level of 1.45 mg/dl (baseline creatinine level 0.9 mg/dl), urea, 73 mg/dl, uric acid, 12.8 mg/dl, estimated glomerular filtration rate 52 ml/minute, hemoglobin 11 mg/dl, and C-reactive protein 0.8 mg/dl. He was treated with an intravenous saline infusion, subcutaneous calcitonin, and intravenous glucocorticoids, followed by intravenous bisphosphonates. Due to his persistent hypercalcemia he was subsequently given 120 mg denosumab (Prolia). During hospitalization, there was significant improvement in his mental status concurrently with the normalization of his calcium level, which was 9.44 mg/dl at discharge.

His past history was remarkable for his 2016 diagnosis of Rai stage IV chronic lymphocytic leukemia. The fluorescence *in situ* hybridization reaction revealed the deletion of the short arm of chromosome 17 (del (17p)) in 20% of B-cell lymphocytes. In addition, in a bone marrow biopsy, the patient was found to have unmutated IGHV gene status and ZAP 70 expression in leukemia cells.

In 09/2017 he was admitted to the Department of Internal Medicine because of autoimmune hemolytic anemia. Treatment with a high dose glucocorticoid was started, followed by a slow tapering down of the dose. In 12/2017 he was again admitted to the Department of Internal Medicine with left hemiparesis, dysarthria, and left facial nerve palsy. Complete right carotid occlusion was found in the CT angiography.

After discharge, he was treated with 2 courses of a Rituximab + Bendamustine protocol with a significant decrease in lymphocytes counts. In September 2018 treatment with the second line agent Ibrutinib was started. One month later, he developed paroxysmal atrial fibrillation with spontaneous conversion to sinus rhythm. Six days before the present admission, the patient was hospitalized because of paroxysmal atrial fibrillation with no evidence of myocardial ischemia. Ibrutinib was stopped because it was presumed to be the cause of the arrhythmia. During his present hospitalization additional laboratory tests revealed an elevated LDH level of 2250 U/l (reference range: 230–480), an alkaline phosphatase level of 220 U/l (reference range: 30–120), and low levels of PTH: 4.8 pg/ml (reference range: 18.4–81), 25 hydroxyvitamin D level of 52 mmol/l (reference range: 75–220) with normal 1.25 dihydroxyvitamin D level of 61.3 nmol/l (reference range: 50–190), WBC 108.000 (reference range: 4.5–11.5 K/μl), Lymphocytes 57%, PLT count of 88.000 (reference range: 150–450 K/μl).

**Figure 1:**
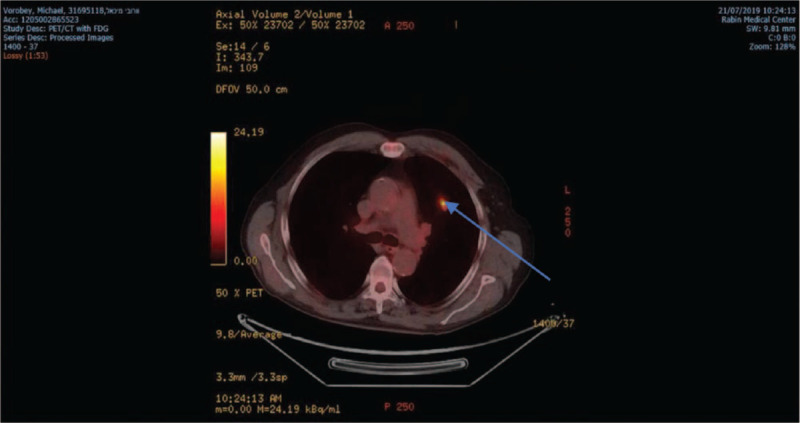
FDG uptake in left upper lobe on PET CT.

In the setting of his elevated LDH level, further decrease in his PLT level to 45.000 and severe hypercalcemia, it was decided to perform a positron emission tomography/computed tomography to assess the possibility of Richter transformation. The scan revealed a 1.3 cm nodule with an intensive uptake of tracer in the upper left lobe of the lung and in vertebra D 7, suspicious of metastasis (Fig. 1).

He underwent a bronchoscopy with a transbronchial biopsy of the lesion. Non-small cell carcinoma displaying positive CK 7 and negative to TTF-1, napsin-A, and p 40 immunostaining was diagnosed. An epidermal growth factor genotype was negative for mutations. Immunohistochemical analysis was negative for ALK and ROS1 protein rearrangement as well as PDL1 expression. He underwent 1 course of chemotherapy in September 2019 that included carboplatin, paclitaxel, and pembroluzumab. Results of the molecular analysis of the tumor were negative for epidermal growth factor receptor (EGFR) genotype mutations, ALK and ROS 1 rearrangement, and PDL-1 protein expression. He then began treatment with venetoclax for his refractory CLL.

Ten days after starting therapy with venetoclax, he was admitted to the internal medicine department with large left upper lobe pneumonia. A chest CT revealed a large infiltrate with an air bronchogram. A subsequent urine antigen test was positive for legionella pneumonia. He completed treatment with respiratory quinolone for 21 days and was returned to oncological and hematological supervision.

The patient received a second course of chemotherapy, but 4 days later he was admitted with suspected pneumonia and despite the broad-spectrum antibiotic treatment, respiratory failure developed and he passed away.

## 3 Discussion

We have presented a patient with severe life-threatening hypercalcemia that occurred a week after withdrawal of ibrutinib treatment. Ibrutinib is a novel, Bruton tyrosine kinase irreversible inhibitor that has demonstrated efficacy in improving survival of older CLL patients with high-risk genetics.^[5]^ The treatment with ibrutinib may cause significant increase in atrial fibrillation events due to structural atrial disorder and impairment in calcium handling in cardiomyocytes.^[5,6,7]^ Ibrutinib treatment has also been suggested as a risk factor for the development of Richter transformation in patients with CLL.^[8]^ In one case report, RT was characterized by severe hypercalcemia, more aggressive course, poorer prognosis and the same molecular features as those of the pre-existent CLL.^[8]^

The 2 main causes of hypercalcemia are primary hyperparathyroidism and cancer.^[3]^ Humoral hypercalcemia of malignancy is responsible for 80% of cases of hypercalcemia related to malignancy and is due to an increase in parathyroid hormone-related protein secretion. The remaining 20% is related to the local destruction of bone by tumor, known as local osteolytic hypercalcemia. Less than 1% of hypercalcemia is related to elevated secretion of 1.25 hydroxyvitamin D and ectopic PTH secretion. Coexistence of cancer and PHPT should be taken into consideration if elevated or incompletely suppressed PTH levels are observed in the patient with suspected humoral hypercalcemia of malignancy.^[9]^ One study shows that about 11% of patients with hypercalcemia and malignancy had concomitant PHPT.^[10]^ The most common type of cancers leading to hypercalcemia are squamous cell tumors, particularly squamous cell lung carcinoma.^[10,11]^ Hematologic malignancies including nonHodgkin's lymphoma, multiple myeloma and, rarely, CLL/small cell lymphocytes lymphoma can cause hypercalcemia, making up 9% of all malignancy-related hypercalcemic disorders.^[9]^ As a rule, PTHrP- mediated hypercalcemia is associated with a poor prognosis and a median survival of only 52 days.^[9]^ Nevertheless, patients with hematologic malignancies had substantially better outcomes with a median survival of 362 days.^[10]^ It is worth noting that no correlation was found between PTHrP or calcium levels and survival.^[10]^

Treatment of severe hypercalcemia includes intensive hydration, calciuresis, and inhibition of osteoclast-induced bone resorption.^[3,4,9,11]^ Intravenous bisphosphonates, preferably with the use of zoledronic acid rather than pamidronate, is the treatment of choice for increased calcium levels related to malignancy.^[10,11,12,13]^

Nevertheless, almost one third of patients with HHM did not respond to, or relapsed after, bisphosphonate treatment.^[11,12]^

Denosumab is a monoclonal antibody reduces the osteoclast-mediated bone resorption due to blocking RANKL.^[4,14]^ In animal models of HHM, osteoprotegerin inhibition of RANKL more effectively decreased calcium levels than zoledronic acid.^[15]^ In a study of 31 patients with solid and hematologic malignancies diagnosed with HCM resistant to bisphosponate treatment, 64% of patients had calcium levels <11.5 mg/dl 10 days after denosumab treatment. In this study, the estimated median response duration of treatment, both complete and partial, was 104 days.^[10]^

The patient under discussions was known to have a high risk CLL and exhibited a HCM diagnosed on the basis of a low level of PTH, a low normal level of 25 (OH) vitamin D and 1.25 (OH) vitamin D. A non-small cell carcinoma of lung with solitary bone metastasis in vertebra D7 was discovered after extensive investigation of his severe symptomatic hypercalcemia.

A small number of CLL patients with hypercalcemia related to PTHrP have been described,^[16–21]^ including cases of CLL related to Richter transformation.^[22,23]^ Interestingly, in contrast to our patient, most of these subjects relapsed with increased lymphocytes counts. Our patient developed hypercalcemia without significant elevation of his lymphocytes count but a large percentage of atypical cells were found in his blood smear. To best of our knowledge, only 1 previous report described a patient presenting with severe hypercalcemia, who was diagnosed with a coexistent renal cell carcinoma and diffuse large B-cell lymphoma.^[24]^

## 4 Conclusion

As far as we have been able to ascertain, this is only the second case of 2 different malignancies presenting in the same patient, either of which could be responsible for his developing HHM. We speculate that the coexistence of different types of tumor cells could potentiate the severity and negative effects of hypercalcemia. This case highlights the importance of close attendance to all details related to a patient's presenting illness.

## Acknowledgments

The authors would like to dedicate this report to the memory of our patient who battled his illness with gratitude and acceptance.

We thank Nancy Peled for the English editing of the manuscript.

## Author contributions

**Conceptualization:** Shay Brikman, Guy Dori.

**Data curation:** Dafna Chap Marshak.

**Writing – original draft:** Elena Chertok Shacham.

**Writing – review & editing:** Avraham Ishay.
